# Prospective economic evaluation of a predictive artificial intelligence model for sepsis: Effects on hospital costs and return on investment

**DOI:** 10.1371/journal.pgph.0006059

**Published:** 2026-08-31

**Authors:** Andres Giglio, Eric Macias-Fassio, Santiago Salas-Sosa, David Lopez, Cristina Pruenza, Aythami Morales, Marcio Borges-Sa

**Affiliations:** 1 Multidisciplary Sepsis Unit, Son Llatzer Hospital, Palma, Spain; 2 Multidisciplinary Sepsis Group, Health Research Intitute of the Balearic Islands (IdISBa), Palma, Spain; 3 University of the Balearic Islands, Palma, Spain; 4 Fundación Código Sepsis, Valencia, Spain; 5 Faculty of Medicine, Finis Terrae University, Santiago, Chile; 6 Critical Care Center, Clínica Las Condes Hospital, Santiago, Chile; 7 BiometricsAI, Universidad Autónoma de Madrid, Madrid, Spain; 8 Instituto de Ingeniería del Conocimiento, Madrid, Spain; 9 Departamento de Matematicas, Universidad de las Palmas de Gran Canaria, Canaria, Spain; University of Antwerp, BELGIUM

## Abstract

Early sepsis detection is essential for improving outcomes and reducing costs, but traditional rule-based systems have limited accuracy and real-world evidence for machine-learning alternatives remains scarce. In this context, BIAlert-Sepsis predicts sepsis risk within 24 hours using historical hospital data, and evaluating its implementation in a tertiary hospital setting provides an opportunity to quantify its clinical benefits and economic value. We conducted a retrospective quasi-experimental before–after study including all septic patients admitted from January 2011 to June 2024. The baseline period (Jan 2011 – Mar 2019) was compared with the AI implementation period (Apr 2019 – June 2024), excluding the COVID-19 interval. Outcomes were assessed using adjusted generalized linear models and interrupted time series regression. A hospital-perspective economic evaluation incorporated implementation and maintenance costs, and a 5-year model estimated net benefit and return on investment (ROI), supported by deterministic and probabilistic sensitivity analyses. A total of 8,039 patients were included (6,168 baseline period; 1,871 AI period). Demographic and clinical characteristics were comparable across periods. During the AI period, ICU admissions decreased from 34.4% to 30.4% (adjusted p = 0.001), accompanied by significant reductions of 0.35 ICU days and 0.59 ward days per patient. Mean admission costs declined from 26,517€ to 24,630€ (adjusted p = 0.005). After covariate adjustment, AI implementation was associated with a 26.1–31.1% reduction in mean admission costs across GLM models. Interrupted time series analysis identified a modest immediate cost level change after AI implementation and a larger sustained decline during the post-COVID period. The 5-year economic model projected a cumulative discounted net benefit of 3.55M€ and a 528% ROI. BIAlert-Sepsis was associated with favourable clinical outcomes and lower costs, with economic modelling suggesting early breakeven and positive financial returns.

## Author summary

Sepsis is a life-threatening condition in which early detection is critical to improving survival and reducing healthcare costs. However, traditional rule-based screening systems often fail to identify high-risk patients early enough. AI offers a promising way to support earlier and more accurate detection.

In this study, we evaluated BIAlert-Sepsis, a ML system designed to predict sepsis risk up to 24 hours before clinical recognition. We analysed data from more than 8,000 septic patients admitted to a tertiary hospital, comparing clinical outcomes and hospital costs before and after AI implementation. The AI system was associated with fewer intensive care unit admissions, shorter hospital stays, and significantly lower patient costs. A five-year economic projection showed that the system generated substantial financial savings and a strong return on investment.

These findings provide real-world evidence that AI-based sepsis detection may help improve hospital efficiency while reducing costs, supporting the integration of predictive AI tools into routine clinical care.

## Introduction

Sepsis, defined as life-threatening organ dysfunction caused by a dysregulated host response to infection, remains a leading cause of global mortality [1]. In 2017, an estimated 48.9 million incident cases and 11 million sepsis-related deaths occurred worldwide, representing 19.7% of all global deaths [2]. Beyond its clinical burden, sepsis imposes substantial economic costs, with average hospital expenditure per patient ranging from 17,158€ to 53,349€ across different countries, accounting for approximately 2.65% of national healthcare budgets [3]. The clinical heterogeneity of sepsis presentations is compounded by lack of consensus on diagnostic criteria. European hospitals demonstrate considerable variability in their approaches: 45.4% employ Sepsis-3 definitions, 24.5% apply Sepsis-1, and 23.2% use both concurrently [4]. This diagnostic inconsistency both reflects and perpetuates the heterogeneity challenge, as different criteria capture different patient phenotypes and stages of illness severity. Delayed recognition and treatment initiation carry severe prognostic implications—inappropriate initial antimicrobial therapy reduces survival from 52% to 10.3%, representing a five-fold decrease [5]. Consequently, there is urgent need for detection systems capable of identifying sepsis across diverse clinical presentations, diagnostic frameworks, and hospital settings.

Traditional rule-based detection tools—including SIRS, SOFA, and qSOFA—demonstrate significant limitations in sensitivity, specificity, and overall accuracy, generating excessive false-positive alerts that contribute to clinician alert fatigue and reduced responsiveness [6–8]. Machine learning (ML) approaches have emerged as a strategy to address these limitations, showing variable but generally superior performance compared to traditional scoring systems [9–13]. However, most Artificial Intelligence (AI) studies rely on retrospective administrative coding rather than prospective clinical validation [14] and focus on single settings rather than hospital-wide surveillance. In this context, we developed BIAlert-Sepsis, a hospital-wide ML system based on prospective case validation [15], approved by the Spanish Agency of Medicines and Medical Devices as a class IIa medical device.

Despite these advances, the economic impact of AI-based sepsis detection remains inadequately characterised. Published cost analyses have relied predominantly on modelling exercises rather than observed implementation data [16,17]. Studies reporting real-world implementation outcomes have either evaluated AI algorithms without formal economic analysis [18] or assessed sepsis protocols without AI augmentation [19]. The incremental economic benefit of AI in established sepsis programmes remains unquantified.

This study represents one of the first real-world economic evaluations of AI-based sepsis detection using implementation data. The primary objective was to evaluate five-year return on investment (ROI) associated with BIAlert-Sepsis implementation. Secondary objectives included assessment of per-patient cost impact and changes in hospital resource utilisation (ICU days, ward days, and readmissions).

## Materials and methods

### Study design and setting

We conducted a retrospective quasi-experimental before–after study to evaluate the clinical and economic impact of BIAlert-Sepsis, an AI-based system for early sepsis and septic shock prediction implemented at Hospital Son Llàtzer (HSLL) [15]. This evaluation took place in a hospital with a long-standing and mature sepsis-management infrastructure. HSLL is a 450-bed tertiary care hospital in Spain serving a catchment population of approximately 300,000 inhabitants. The centre is equipped with an 18-bed multidisciplinary ICU and advanced laboratory and informatics infrastructure, enabling seamless integration of AI-driven clinical decision support. A formal multidisciplinary sepsis code has been in place since 2006, ensuring systematic, prospective assessment and confirmation of all suspected cases by trained clinicians. In addition, since 2011 the hospital has operated a rule-based system that continuously flagged patients at risk of sepsis using predefined clinical criteria [20]. BIAlert-Sepsis was fully integrated into the electronic health record (EHR) in March 2019, following a one-month period of system adaptation and staff training.

The study compared outcomes between a baseline period (January 2011–March 2019), during which the rule-based detection system was in use, and the AI implementation period (April 2019–June 2024). Data corresponding to the COVID-19 pandemic (March 2020–October 2022 [21]) were excluded to avoid bias due to major disruptions in care delivery, patient mix, and resource use. The analysis was carried out from a hospital perspective, incorporating direct healthcare costs associated with inpatient care as well as the costs of implementing and maintaining the AI-based system. All methodological choices adhered to current best practices for health economic evaluations and followed the CHEERS 2024 reporting guidance, ensuring transparency in model assumptions, parameter selection, uncertainty characterisation, and analytical steps [22].

### Ethics statement

The study was approved by the Ethics and Health Research Committee of the Balearic Community (CEIC-Ib; approval ID 463721), which granted a waiver of informed consent because of the retrospective nature of the study. Following review by the Legal Departments of the hospital and IdISBa and by the Hospital Research Commission, authorisation to conduct the study was obtained from the hospital management. The study was conducted in accordance with applicable European and Spanish data-protection legislation.

### Population and selection criteria

The source population comprised all hospital admissions to the sepsis protocol between 2011 and 2024 with complete clinical and administrative records. Patients >= 14 years in whom the hospital sepsis protocol was activated were included. Admissions during the COVID-19 period (March 2020–October 2022) and those with incomplete or inconsistent data were excluded from the analysis. Data were accessed for research purposes between 01/11/2024 and 15/11/2024.

The baseline period (2011–2019) included *N*_1_ = 6,168 septic patients, representing 3.5% of 173,293 admissions. The AI implementation period (2019–2024) included *N*_2_ = 1,871 septic patients, likewise representing 3.5% of 53,113 admissions. These proportions indicate consistent sepsis incidence across periods, supporting comparability between cohorts.

### Variables and data sources

Demographic and clinical variables included age, sex, limitation of therapeutic effort (LTE), admission to ICU, ICU readmission and hospital readmission within 30 days. Resource utilisation variables comprised length of stay (LOS) in ICU, and in non-critical care units (ward). The discharge service type (medical, surgical, or critical care) was recorded to apply corresponding unit costs.

### Intervention: AI-based sepsis prediction system

BIAlert-Sepsis is an AI system that employs ML algorithms trained on historical hospital data to predict patients at high risk of developing sepsis within the following 24 hours [15]. The system delivers real-time alerts to clinicians through the EHR interface. Model performance was continuously monitored using Area Under the Curve (AUC), sensitivity, specificity, positive predictive value, negative predictive value, and clinical decision-making protocols were standardised to ensure consistent response to alerts.

### Clinical and economic outcomes

Clinical effectiveness was assessed through three key outcomes: ICU admission rate, LOS in ICU and LOS in Ward.

For the economic evaluation, the cost per admission was estimated using the officially assigned daily reimbursement values established by the public payer of the Balearic Islands for 2025 (IB Salut). These standardized tariffs correspond to the designated type of bed (critical care, medical ward, or surgical ward) and reflect the comprehensive daily care provided to patients. Because reimbursement-based tariffs reflect average rather than marginal costs, reductions in length of stay may not translate into proportional short-term cash-flow savings for the hospital, particularly when freed capacity is filled by other admissions or when most overhead costs remain fixed. Accordingly, these estimates should be interpreted as an upper-bound approximation of the economic value attributable to AI implementation.

All outcomes and costs were compared between the baseline (2011–2019) and AI-implementation (2019–2024) periods, excluding the COVID-19 pandemic years (2020–2022).

### Statistical analysis

Continuous variables were summarized as means with interquartile ranges (IQRs), and categorical variables as frequencies and proportions. Differences between study periods were evaluated using the Wilcoxon rank-sum test for continuous variables and the Chi-square test for categorical variables.

For the primary adjusted comparison of mean cost per patient between the baseline period (2011–2019) and the AI implementation period (2019–2024), we fitted a generalized linear model (GLM) with a Gamma distribution and log link function to account for the right-skewed distribution of hospitalization costs. The model adjusted for age, sex, ICU admission, ICU readmission, limitation of therapeutic effort (LTE), organ dysfunction, month and year.

ICU admission and ICU readmission were included because they are major drivers of hospitalization costs in sepsis care and explain a substantial proportion of variability in total cost. At the same time, these variables are clinically plausible intermediate outcomes that may be affected by earlier sepsis detection and treatment. Specifically, AI implementation may reduce costs partly through earlier recognition and treatment, leading to lower ICU utilisation and, consequently, reduced hospitalization costs.

To address this causal structure, we distinguished between two complementary estimates. First, the model including ICU admission and ICU readmission was interpreted as estimating a more conservative direct effect of AI implementation on cost, conditional on ICU utilisation. Second, a reduced model excluding ICU admission and ICU readmission was interpreted as estimating the broader total effect, allowing cost differences mediated through changes in ICU use to be captured.

Accordingly, we specified two GLMs: (i) a full model including ICU admission and ICU readmission, defined as the direct-effect model and used as the primary adjusted analysis; and (ii) a reduced model omitting ICU admission and ICU readmission while retaining all non-mediator covariates, defined as the total-effect model. Presenting both models allowed us to evaluate whether the estimated economic impact of AI implementation was robust to alternative assumptions about the role of ICU utilisation in the causal pathway.

The primary direct-effect model was specified as follows:


$$\begin{array}{cc} \log(E[{\text{Mean Cost}}_{i}])=\; & \beta_{0}+\beta_{1}{\text{Periods}}_{i}+\beta_{2}{\text{Age}}_{i}+\beta_{3}{\text{Sex}}_{i}\\ & +\beta_{4}{\text{ICU Admission}}_{i}+\beta_{5}{\text{ICU Readmission}}_{i}\\ & +\beta_{6}{\text{LTE}}_{i}+\beta_{7}{\text{Dysfunction}}_{i}+\beta_{8}{\text{Year}}_{i}+\beta_{9}{\text{Month}}_{i} \end{array}$$

To assess longitudinal trends and the temporal effect of the AI-based sepsis prediction system, we performed an interrupted time-series (ITS) analysis using ordinary least squares (OLS) segmented regression [23,24]. The model included separate level and slope parameters for each intervention point (AI implementation, COVID-19 onset, and post-COVID period), as well as a continuous time variable to capture underlying trends. Assessment of residuals with the Durbin–Watson (DW) statistic revealed slight temporal autocorrelation (DW = 1.6). To account for this, we applied Newey–West HAC standard errors, which adjust for heteroscedasticity and autocorrelation to ensure robust inference. The mathematical formulation is provided in S1 Appendix in S1 File.

All statistical analyses were performed using Python, with two-sided p-values < 0.05 considered statistically significant after adjustment for multiple testing using the Holm method.

### Patient costs

The cost of the index episode was calculated as:


$$\begin{array}{c} \text{Total Cost}=(\text{Days in ICU}\times C_{\text{ICU}})+(\text{Days in Ward}\times C_{\text{Ward}}) \end{array}$$

where *C*_ICU_ = 2,094€ represents the average daily hospitalization cost in the intensive care unit, and *C*_Ward_ corresponds to the average daily cost in non-critical care units, which ranged between 1,092€ and 1,394€ depending on the type of service. Thirty-day readmissions were identified by matching patient identification codes and summing the corresponding readmission costs. The total cost per patient was defined as the sum of the index episode cost and the readmission costs. Unit costs were expressed in euros and standardized to 2025 price levels to ensure comparability across study periods. The use of a single price year for all admissions, rather than year-specific tariffs, was a deliberate methodological choice to isolate between-period differences in clinical resource use, including ICU and ward length of stay and ICU admissions. Using year-specific tariffs could have introduced exogenous price variation unrelated to the AI intervention, such as annual tariff revisions, salary adjustments, or pharmaceutical and energy price fluctuations, thereby confounding comparisons between the baseline and AI periods. By holding unit costs constant at 2025 levels, observed cost differences more directly reflect changes in resource utilisation rather than administrative pricing effects.

### Estimation of savings per patient

Cost savings per patient were calculated by multiplying the reductions in hospital resource use by their corresponding unit costs. Savings encompassed avoided expenditures from shorter ICU and ward stays, as well as decreases in ICU admissions. Each reduction component was paired with its specific unit cost, and the sum represented the average per-patient saving.

Unit costs were obtained from the hospital financial data and updated to 2025 euros according to the official reimbursement values established by IB Salut:

2,094€ per ICU day, 1,243€ per non-ICU (ward) day, 8,773€ per ICU admission on average. Accordingly, per-patient savings were computed as follows:


$$\begin{array}{c} \text{Savings per patient}=(\Delta_{\text{ICU}}\times C_{\text{ICU}})+(\Delta_{\text{ward}}\times C_{\text{ward}})+(\Delta_{\text{adm}}\times C_{\text{adm}}) \end{array}$$

where $\Delta_{\text{ICU}}$ and $\Delta_{\text{ward}}$ represent the mean reduction in ICU and non-ICU length of stay (days), respectively, and $\Delta_{\text{adm}}$ correspond to reductions in ICU admission rate.

The IB Salut bed-day tariffs applied in this analysis correspond to official all-inclusive per diem reimbursement rates published by the regional health authority, each representing a single consolidated price per day of stay that integrates all components of inpatient care (personnel, pharmacy, diagnostics, services, and overheads) and is therefore not further disaggregated.

### Economic modelling of return on investment

An economic model was developed to estimate the return on investment (ROI) and the net present value (NPV) associated with the implementation of the predictive AI system for sepsis. The annual benefit was calculated as:


$$\begin{array}{c} \begin{array}{cc} \text{Annual benefit} & =(\text{Savings per patient})\times (N_{\text{septic patients}})\\ & \times (\text{AI adoption ramp})\times (\text{System accuracy}) \end{array} \end{array}$$

In the base-case analysis, system accuracy was set at 0.85. This value corresponds to the post-deployment performance metrics reported for BIAlert-Sepsis by the developer and is consistent with AUC values reported for prospectively validated machine-learning sepsis classifiers in the published literature [10,15]. Adoption of the AI system was modelled using a progressive effectiveness ramp, assuming 60% adoption in the first year, 80% in the second and third years, and full adoption (100%) in years four and five. This schedule reflects the observed pace at which clinicians integrated alerts into routine workflow at HSLL during the first years following go-live.

In addition, a pessimistic scenario was analysed using a lower system accuracy value of 0.65 and a more conservative adoption schedule. This scenario assumed a slower implementation ramp of 40%, 60%, 80%, 80%, and 100% over years 1–5, respectively. A 3% annual discount rate was applied to all future financial flows, in accordance with standard economic evaluation practice [25].

The annual cost of the AI system was defined as the sum of the one-time installation cost and the recurring annual maintenance cost:


$$\begin{array}{c} \text{Annual cost}=\text{Installation cost}+\text{Maintenance cost} \end{array}$$

The installation cost (200,000€) covers the deployment of the microservices architecture underpinning the BIAlert-Sepsis platform, including its integration with the hospital information system. The annual maintenance cost (100,000€) covers the continuous operational support of the platform, comprising the analysis of resource utilisation, the monitoring of alert volumes and model performance metrics, and model re-training when required to mitigate performance drift.

Economic performance was evaluated using standard financial indicators. The NPV represents the discounted net benefit over the study horizon, and ROI (%) expresses the relative gain per unit cost:


$$\begin{array}{c} \begin{array}{cc} NPV & =\sum_{t=0}^{T}\frac{{\text{Benefits}}_{t}-{\text{Costs}}_{t}}{(1+r{)}^{t}},\\ ROI(\%) & =\frac{\text{Total benefits}-\text{Total costs}}{\text{Total costs}}\times 100 \end{array} \end{array}$$

where *r* denotes the annual discount rate and *T* the time horizon (in years) of the evaluation.

### Sensitivity analysis

A sensitivity analysis was conducted to explore uncertainty in the economic model parameters. First, a one-way deterministic sensitivity analysis was performed, in which each key parameter was varied individually across its 95% confidence interval (CI) while holding all other parameters at their base-case values. The impact of these variations on the net present value was summarized using a tornado diagram. To quantify the combined impact of parameter uncertainty on the economic evaluation, we performed a probabilistic sensitivity analysis (PSA) using a Monte Carlo simulation with 10,000 iterations [26]. In each iteration, all key model parameters were simultaneously drawn from predefined probability distributions. For every simulated parameter set, the net present value (NPV) and return on investment (ROI) were recalculated. For cost inputs, the standard error was assumed to be 20% of their respective mean values [27]. For both NPV and ROI, we report the mean together with its 95% CI, estimated using bootstrap resampling of the simulated PSA outputs. Details on parameter values and distributional assumptions are provided in S2 Appendix in S1 File.

## Results

### Descriptive characteristics of the study population

As shown in Table 1, demographic characteristics were comparable between groups, with no significant differences in age (mean 65.8 vs. 65.4 years; adjusted p = 0.624), sex distribution (40.9% vs. 42.5% female; adjusted p = 0.624), organ dysfunction (1.7 vs. 1.8; adjusted p = 0.222), or shock criteria (8.6% vs. 8.9%; adjusted p = 0.708). These findings indicate similar case-mix and clinical complexity between the baseline and AI implementation periods.

**Table 1:** Descriptive and statistical comparisons between the baseline and AI periods.

Variable	Baseline period	AI implementation	p-value	Holm
**ICU admission**	2,122 (34.4%)	569 (30.4%)	<0.001	**0.001**
**ICU LOS**	12.2 (10.0–13.3)	11.85 (10.0–13.0)	<0.001	**0.001**
**Admission cost**	26,517.2 (7,644–29,316)	24,630.2 (7,509–26,486)	0.001	**0.005**
**Ward LOS**	15.4 (6.0–18.0)	14.81 (5.0–16.0)	0.006	**0.047**
Organ dysfunction	1.7 (1.0–2.0)	1.8 (0.0–3.0)	0.037	0.222
30-day readmission	278 (4.5%)	56 (3.0%)	0.027	0.189
LTE	1,152 (18.7%)	310 (16.6%)	0.117	0.585
ICU readmission	155 (2.5%)	35 (1.9%)	0.130	0.585
Sex (female)	2,520 (40.9%)	795 (42.5%)	0.218	0.624
Age (years)	65.8 (55.0–79.0)	65.4 (56.0–78.0)	0.312	0.624
Hypotension/Lactate	528 (8.6%)	166 (8.9%)	0.708	0.708

**Fig 1:**
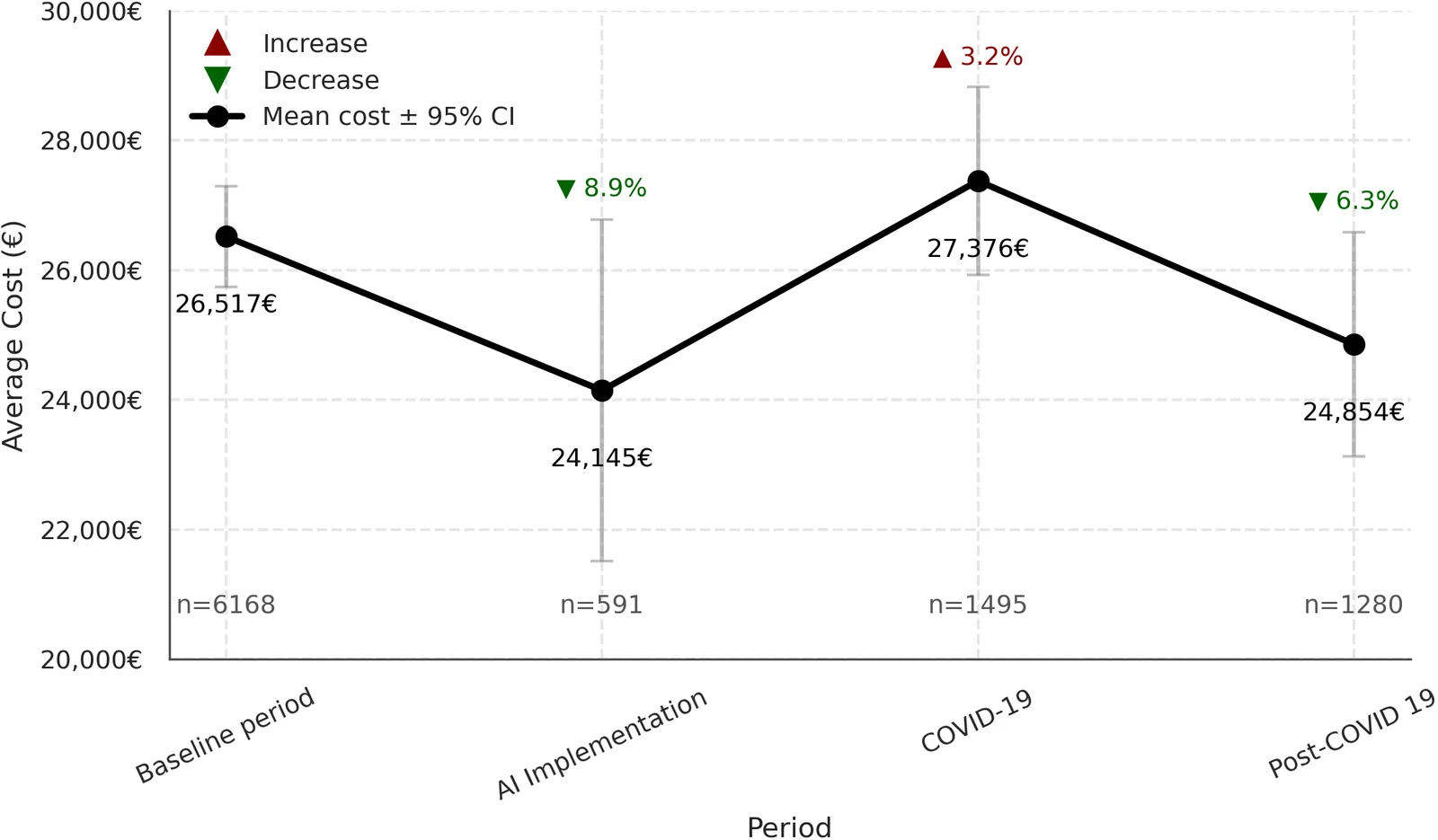
Average cost per patient (€) across study periods. Percentage changes represent increases or decreases compared with the baseline period.

During the AI implementation period, several clinical and economic indicators showed improvement. ICU admissions declined from 34.4% to 30.4% (adjusted p = 0.001). ICU LOS was significantly reduced (mean 12.2 to 11.85 days; adjusted p = 0.001), and ward LOS also decreased (15.4 to 14.81 days; adjusted p = 0.047). ICU readmissions decreased from 2.5% to 1.9%, 30-day readmissions from 4.5% to 3.0%, and the proportion of patients with LTE declined from 18.7% to 16.6%, although none of these differences remained statistically significant after adjustment. Overall, mean admission costs were significantly lower during the AI period (26,517€ to 24,630€; adjusted p = 0.005). Following AI implementation, per-patient savings were associated with reductions in ICU days, ward days, and ICU admissions, yielding an average savings of approximately 1,801€ per patient. Fig 1 shows the temporal evolution of mean admission cost per patient across the study periods. Costs were lower in the AI period than baseline (p = 0.001).

### Adjusted cost analysis

To rigorously assess whether the observed cost reductions during the AI implementation period persisted after adjusting for potential confounders, including patient characteristics and temporal trends, we fitted a generalized linear model (GLM) with a gamma distribution and log link (Table 2). The model showed good explanatory power (Pseudo R^2^ = 0.2881; n = 8,039), indicating that approximately 29% of the cost variability was explained by the included variables. After adjusting for potential confounders, including patient age, sex, ICU admission, LTE, and ICU readmission, the AI Implementation period remained significantly associated with a 26.1% reduction in mean admission cost (Exp[Coef.] = 0.739; p < 0.01). ICU admission remained the strongest cost driver, increasing average cost by 182% (Exp[Coef.] = 2.825; p < 0.01), while ICU readmission was associated with a 116% increase (Exp[Coef.] = 2.165; p < 0.01). LTE increased costs by 30.9% (Exp[Coef.] = 1.309; p < 0.01). Age, sex and dysfunction were not significant predictors of cost (p > 0.05). Several month indicators reached significance, reflecting seasonal fluctuations, and annual cost increased 4.7% over time (Exp[Coef.] = 1.047; p < 0.01). These results indicate that AI implementation was associated with lower admission costs after adjustment for measured patient characteristics and clinical events.

**Table 2:** Gamma GLM (log link) estimating predictors of total admission cost.

Variable	Coef.	Std. Error	p-value	Exp(Coef.)	% Change
Month (2)	0.0885	0.052	0.086	1.093	+9.3%
Month (3)	-0.0043	0.051	0.934	0.996	-0.4%
Month (4)	0.0546	0.054	0.308	1.056	+5.6%
Month (5)	0.0294	0.052	0.575	1.030	+3.0%
Month (6)	0.0548	0.055	0.322	1.056	+5.6%
Month (7)	0.0496	0.055	0.371	1.051	+5.1%
Month (8)	**0.1248**	0.057	**0.028**	**1.133**	**+13.3%**
Month (9)	0.1006	0.055	0.066	1.106	+10.6%
Month (10)	0.0788	0.055	0.151	1.082	+8.2%
Month (11)	0.0678	0.053	0.201	1.070	+7.0%
Month (12)	0.0295	0.052	0.570	1.030	+3.0%
**AI implementation**	**-0.3026**	0.042	**<0.01**	**0.739**	**-26.1%**
Age (years)	-0.0003	0.001	0.665	0.9997	-0.03%
Sex (female)	-0.0267	0.023	0.252	0.974	-2.6%
ICU admission	**1.0384**	0.026	**<0.01**	**2.825**	**+182%**
LTE	**0.2701**	0.031	**<0.01**	**1.309**	**+30.9%**
Dysfunction	-0.0021	0.009	0.811	0.998	-0.2%
Year	**0.0463**	0.005	**<0.01**	**1.047**	**+4.7%**
ICU readmission	**0.7723**	0.077	**<0.01**	**2.165**	**+116%**
**Model summary**					
Number of observations				8,039	
Residual degrees of freedom				8,019	
Model degrees of freedom				19	
Log-Likelihood				-88,447	
Deviance				5,543.8	
Pseudo *R*^2^ (Cragg–Uhler)				0.288	

In the complementary total-effect specification (omitting ICU admission and ICU readmission while retaining all non-mediator covariates, S3 Appendix in S1 File) the AI period coefficient was −0.373 (Exp[Coef.] = 0.689; 95% CI: 0.618–0.768; p < 0.001), corresponding to a 31.1% reduction in mean admission cost. This reduction was larger than the 26.1% estimated in the direct-effect model, as expected when ICU-related variables are not adjusted for. The total-effect model therefore captures both the direct association between AI implementation and cost, and the additional cost reduction potentially mediated through lower ICU admission or readmission.

### Interrupted time series analysis

**Fig 2:**
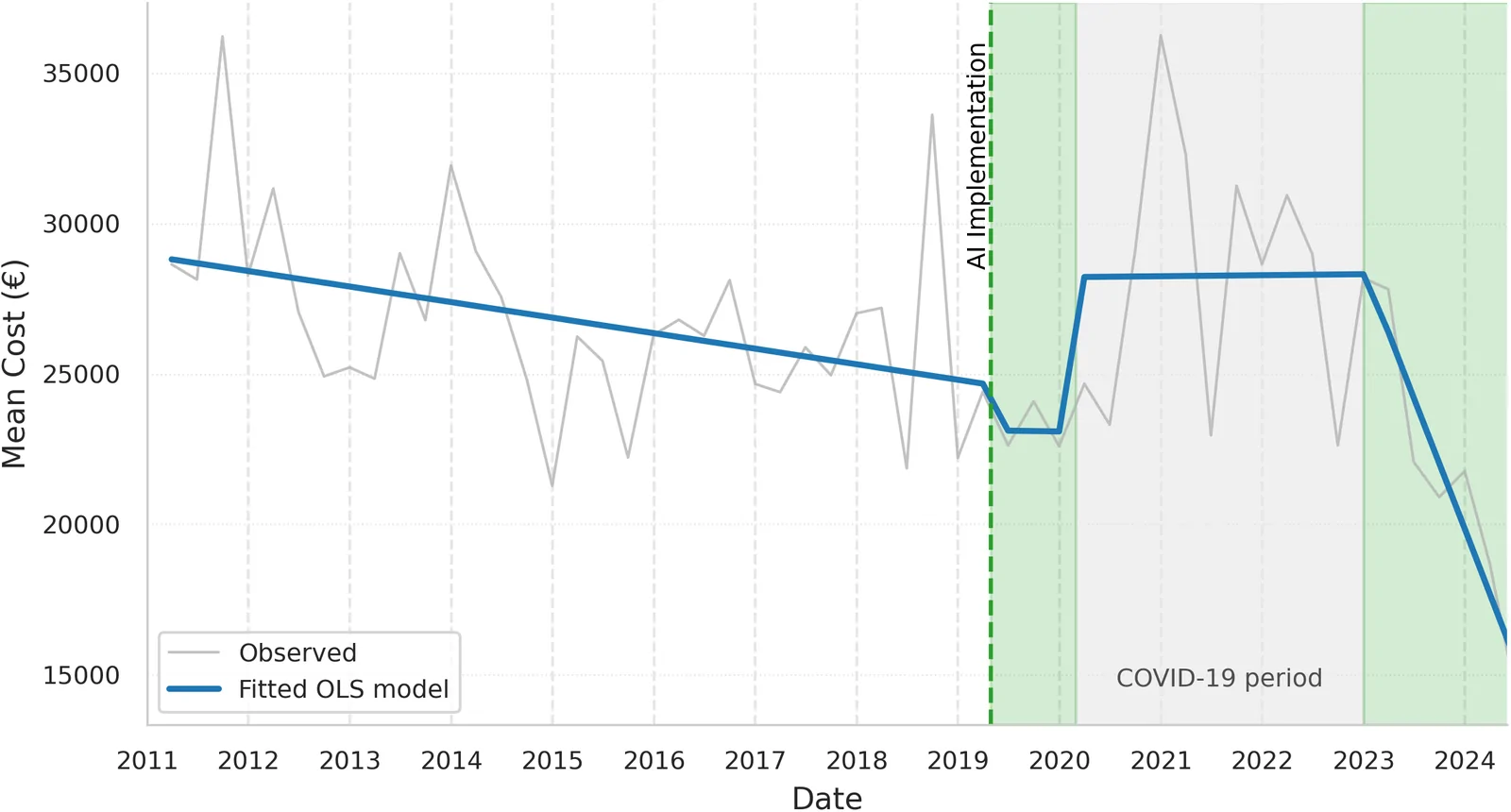
ITS analysis of quarterly mean hospital admission costs, showing observed values and the fitted segmented regression model.

To examine whether the implementation of the AI system was associated with structural changes in quarterly cost trajectories, beyond those explained by routine temporal variation, we conducted an interrupted time series (ITS) analysis using OLS segmented regression with HAC-robust standard errors, modelling both immediate level changes and post-intervention trend changes. Table 3 and Fig 2 summarize the ITS analysis assessing the association between the periods and quarterly mean patient cost. Prior to any intervention, costs already exhibited a statistically significant downward secular trend in mean hospital cost (–129.10€ per quarter; p = 0.001). This pre-existing trend is consistent with progressive improvements in sepsis care at HSLL pre-AI deployment (the multidisciplinary sepsis code dates from 2006 and the rule-based detection system from 2011). Following AI implementation, the model estimated a borderline significant immediate level change (–1,550.49€; p = 0.05) and no meaningful change in trend (+115.76€ per quarter; p = 0.394). At the onset of the COVID-19 period, the model indicated a large and statistically significant positive level shift (+5,127.73€; p = 0.001) and a small non-significant trend increase (+137.43€ per quarter; p = 0.513), indicating that while COVID-19 produced an immediate escalation in cost, it did not significantly modify the underlying trend in quarterly expenditure. During the post–COVID-19 period, the model estimated a non-significant level change (+258.23€; p = 0.828), while the slope change during this period was large and statistically significant (–2,049.89€ per quarter; p < 0.001), indicating a substantially accelerated decline in mean quarterly costs after the pandemic period ended. This post-COVID downward trend is consistent with the gradual scale-up and full integration of the AI system, but cannot be cleanly disentangled from the simultaneous normalisation of hospital activity following the pandemic; it should therefore be interpreted as an association rather than a direct effect of the AI tool.

**Table 3:** Interrupted time series model results estimating the association between interventions and mean patient cost (OLS with HAC standard errors).

Model component	Estimate	SE	95% CI	p-value
**Baseline trend**	**-129.10**	38.31	-204.19 to -54.01	**0.001**
**AI implementation**				
Level change (post-AI)	**-1,550.49**	803.32	-3,124.96 to 23.98	**0.05**
Slope change (post-AI)	115.76	135.75	-150.30 to 381.82	0.394
**COVID-19 onset**				
Level change (post-COVID onset)	**5,127.73**	1,615.07	1,962.25 to 8,293.21	**0.001**
Slope change (post-COVID onset)	137.43	210.26	-274.68 to 549.54	0.513
**Post–COVID-19 period**				
Level change (post-COVID period)	258.23	1,188.26	-2,070.71 to 2,587.17	0.828
Slope change (post-COVID period)	**-2,049.89**	110.21	-2,265.88 to -1,833.89	**<0.001**

### Base-case scenario

The intervention was associated with substantial modelled economic benefits. To evaluate its financial impact, we simulated a 5-year implementation scenario using the cost-savings obtained in Section Patient costs. Table 4 presents the annual costs, annual benefits, net benefit, ROI and the discount factors used to calculate the cumulative discounted net benefits. The full set of parameter values used in this scenario is provided in S2 Appendix in S1 File.

**Table 4:** Annual economic impact of the AI implementation, including discounting and cumulative discounted net benefit (€).

Year	Benefits	Costs	Net	Disc.	Disc. Net	Cumulative	ROI
1	643,478	300,000	343,478	1.0000	343,478	343,478	114.5%
2	857,970	100,000	757,970	0.9709	735,893	1,079,371	271.8%
3	857,970	100,000	757,970	0.9426	714,460	1,793,831	365.1%
4	1,072,463	100,000	972,463	0.9151	889,941	2,683,772	460.5%
5	1,072,463	100,000	972,463	0.8885	864,021	3,547,793	528.2%

In the first year, the system yielded 343,478€ in net savings despite the initial capital expenditure. As AI adoption increased, annual net benefits rose to approximately 0.97€ million from year 4 onwards. The cumulative net benefit became positive during the first year, indicating that the intervention reached its economic breakeven point early in the evaluation period. When incorporating a 3% discount rate from the second year onward, the analysis showed that cumulative discounted net benefits amounted to 3.55€ million by year 5, yielding a ROI of 528.2%.

Under the pessimistic scenario (system accuracy = 0.65; adoption ramp = 40–60–80–80–100%), the 5-year cumulative discounted net benefit was 2,081,594€, with a final-year ROI of 309.9% (Appendix S4 in S1 File). Breakeven was reached in year 1, with cumulative discounted net benefits remaining positive throughout the projection horizon.

### Sensitivity analysis findings

**Fig 3:**
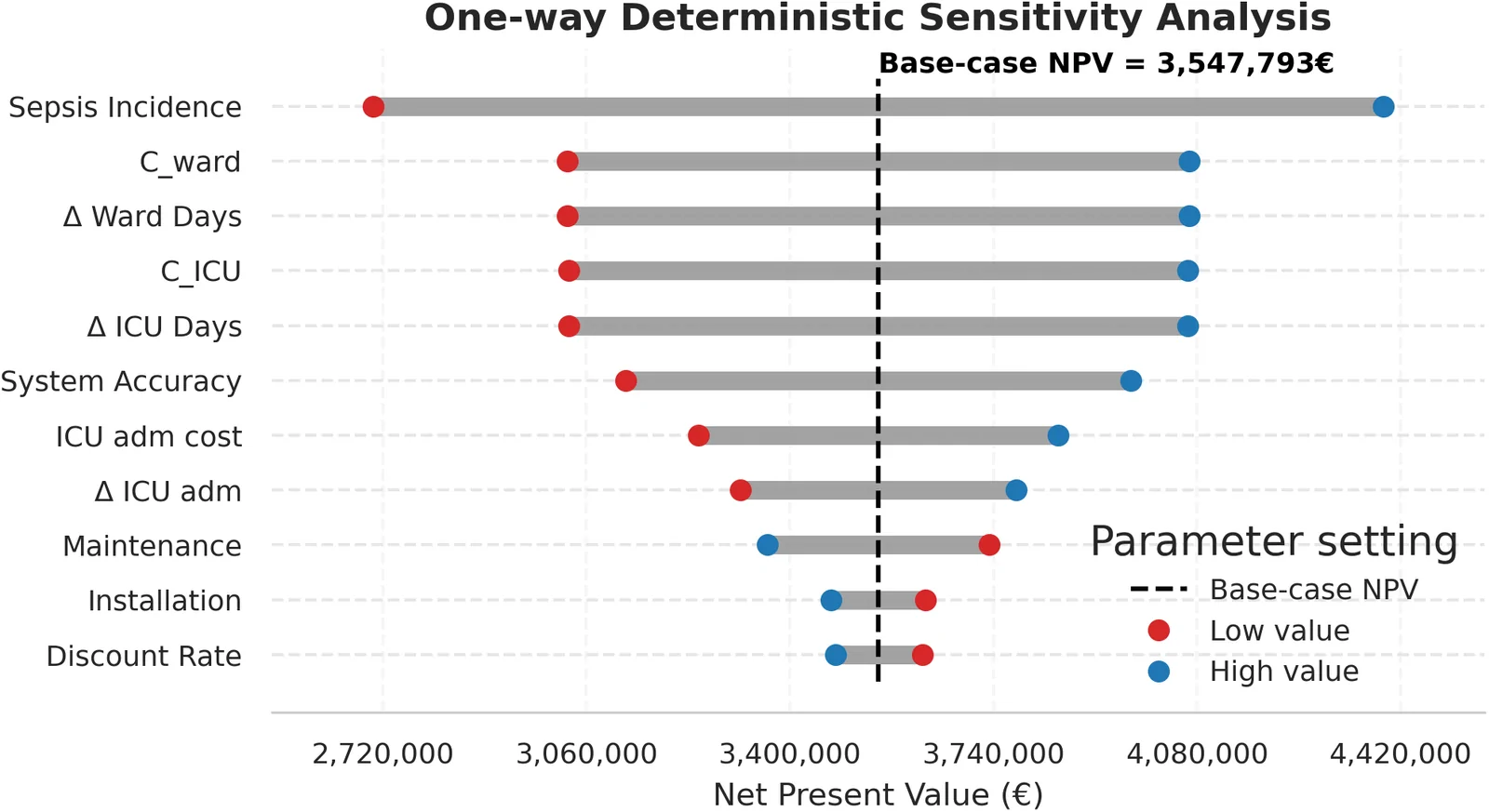
One-way deterministic sensitivity analysis illustrating the impact of key model parameters on the net present value (NPV) of the intervention.

To evaluate the robustness of these economic outcomes, we conducted a one-way sensitivity analysis varying the key model parameters across plausible ranges. This analysis quantified the relative influence of each parameter on the net present value of the intervention. The results are visualized in the tornado plot (Fig 3), which ranks parameters by their impact on the economic outcome. The one-way deterministic sensitivity analysis showed that the model’s NPV was most sensitive to changes in sepsis incidence, which produced the widest variation in economic outcomes. Other influential parameters included ward and ICU costs, length-of-stay reductions, and system accuracy, each generating notable shifts in NPV when varied within their respective ranges. In contrast, maintenance costs, installation costs, and the discount rate had comparatively minor effects on the overall economic results.

**Fig 4:**
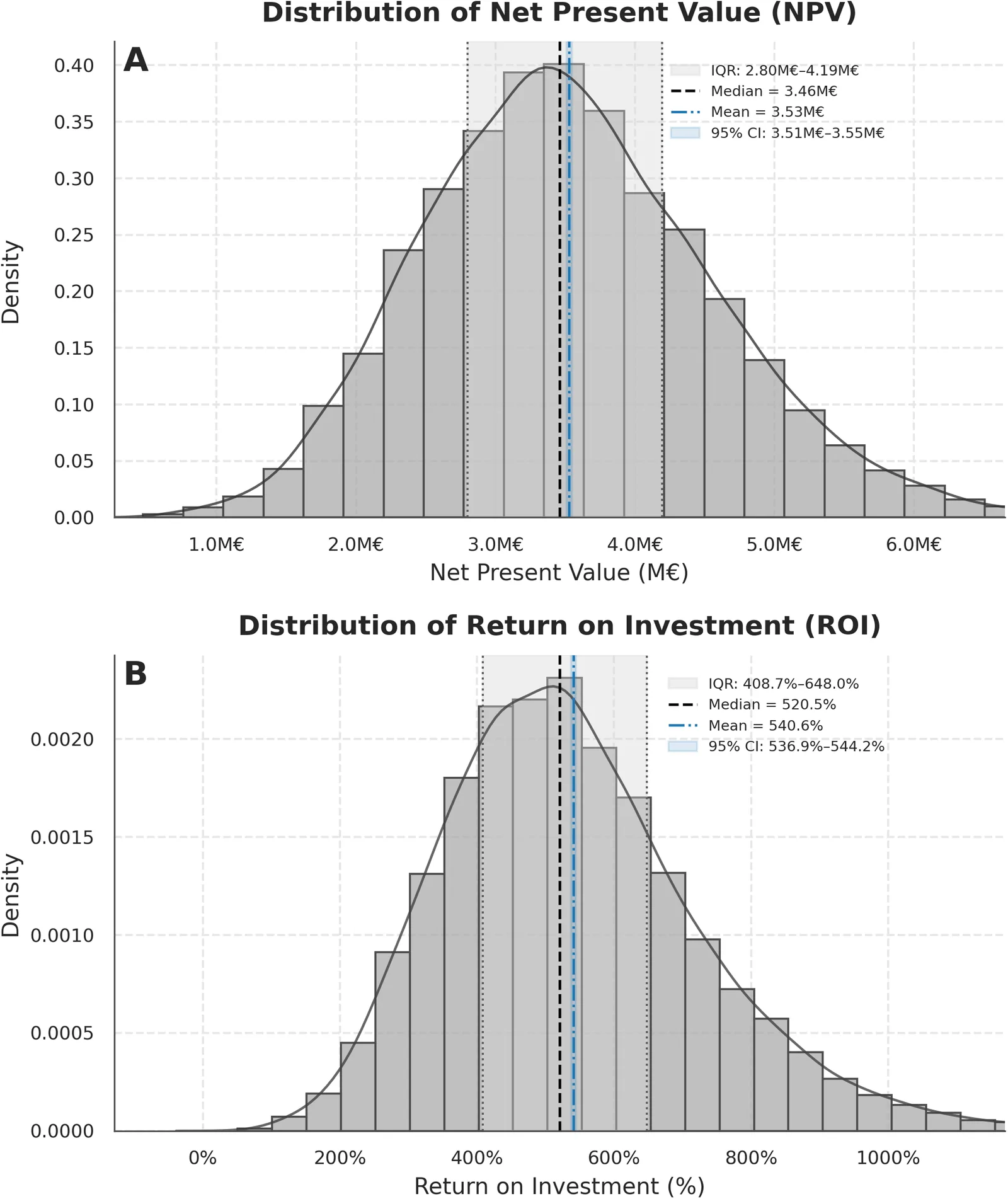
Results of probabilistic sensitivity analysis showing uncertainty in (A) net present value and (B) return on investment across 10,000 Monte Carlo simulations.

To further assess the uncertainty surrounding the economic evaluation, we performed a PSA using a Monte Carlo simulation (Fig 4). Across 10,000 simulations, the intervention remained cost-saving in the large majority of iterations. The distribution of the 5-year discounted NPV had a mean of approximately 3.53€ million, with an interquartile range of roughly 2.80€–4.19€ million and a 95% CI of approximately 3.51€–3.55€ million; fewer than 3% of simulations yielded a negative NPV. Similarly, the ROI had a mean of 540.6% (IQR = 408.7%–648.0%; 95% CI = 536.9%–544.2%). Overall, these results indicate that, under the model’s assumptions and even allowing for considerable uncertainty in clinical and economic parameters, simulations suggest a high probability of positive modelled net benefit.

## Discussion

### Summary of findings

The study included 8,039 septic patients overall (*N*_1_ = 6168 in the baseline period and *N*_2_ = 1871 in the AI period). Initial descriptive comparisons confirmed that demographic and clinical characteristics were well balanced between periods, indicating comparable case-mix distributions. The analysis revealed a significant decline in average costs from 26,517.2€ to 24,630.2€ during the AI implementation period (7.1% reduction). To further examine whether these improvements persisted after accounting for potential confounding factors, we fitted a GLM model with a Gamma distribution and log link. Although unadjusted analyses showed a 7.1% reduction in average admission cost during the AI implementation period, the multivariable GLMs estimated a 26.1% reduction under the direct-effect specification (adjusted for ICU admission and readmission) and a 31.1% reduction under the total-effect specification that did not condition on ICU utilisation.

Building on the adjusted GLM findings, we further examined temporal patterns in costs using an ITS. The ITS analysis should be interpreted in the context of a statistically significant pre-intervention downward trend in quarterly costs (129€ per quarter; p = 0.001), consistent with long-standing quality improvement initiatives at HSLL, including the multidisciplinary sepsis code introduced in 2006 and the rule-based detection system implemented in 2011. These initiatives likely created a strong foundation for subsequent improvements. Following AI implementation, the analysis showed a borderline immediate level decrease (p = 0.05), suggesting a potential early impact of the AI tool, although this finding should be interpreted with appropriate caution. The steeper downward trend observed in the post-COVID period is also temporally aligned with full AI integration and may reflect the added value of AI within an already improving clinical pathway. While the contribution of post-pandemic normalisation cannot be fully excluded, the overall pattern is consistent with AI acting as an important enabling component in sustaining and potentially accelerating cost reductions.

Finally, a base-case economic scenario was simulated to estimate the financial consequences of implementing the AI system over a 5-year horizon. The model projected a substantial cumulative net benefit of 3.55M€ and a positive return on investment (ROI), indicating that the modelled economic scenario suggested early breakeven. Complementary sensitivity analyses (deterministic and probabilistic) confirmed the robustness of these findings. Across all tested parameter ranges and in 10,000 Monte Carlo simulations, the NPV and ROI consistently remained positive, indicating that the economic advantage of the AI system persisted even under conservative assumptions.

These findings indicate that the observed cost savings were not fully explained by measured patient characteristics or clinical complexity and were temporally associated with changes in resource utilisation during the AI implementation phase. Although explaining the cost differences between study periods is complex, one plausible contributing factor may be the greater predictive performance of ML algorithms compared with rule-based tools such as SOFA or qSOFA, an advantage that has been repeatedly demonstrated in the literature [15,28–30].

### Comparison with previous studies

In comparison with prior literature, our findings align with and extend existing evidence regarding the economic benefits of AI-based clinical interventions. Several AI systems for sepsis prediction have been proposed and evaluated, but few have combined real-world implementation with a detailed economic assessment.

Ericson et al. assessed the economic impact of an AI-driven sepsis prediction algorithm capable of identifying sepsis up to three hours before onset and reported a reduction of 0.16 ICU days per patient, corresponding to an average cost saving of 1,009€ per ICU admission [16]. Similarly, Calvert et al. evaluated an AI-based sepsis prediction system in an ICU setting and estimated that its implementation could save approximately $560,000 per year in sepsis-related costs for a 50-bed ICU, primarily driven by reductions in length of stay and improved clinical outcomes [17]. Burdick et al. reported substantial clinical and economic benefits following the implementation of a machine-learning sepsis prediction algorithm. In their evaluation, in-hospital mortality among sepsis-related admissions decreased by 39.5%, hospital length of stay was reduced by 32.3%, and 30-day readmissions declined by 22.7% [18]. In comparison, our study similarly suggests that early sepsis detection may be associated with meaningful economic value, with a robust positive net present value and cost savings driven largely by shortened hospital stays. Unlike prior work, our analysis further integrates implementation, maintenance, and system-level costs, indicating that the intervention remains economically favourable across a wide range of sensitivity parameters, thereby extending the existing evidence base with a more comprehensive assessment of real-world economic outcomes.

### Limitations

Several considerations should be taken into account when interpreting these findings. The quasi-experimental before–after design cannot fully exclude residual confounding or secular trends, despite adjustment for key patient characteristics. However, a stronger comparative design was not feasible, as no other hospital with comparable cost data and a fully functional multidisciplinary sepsis protocol was available. Therefore, this design represented the best possible approach in the present study. The ITS analysis indicated that quarterly costs were already declining before AI implementation, likely reflecting longstanding institutional efforts in sepsis management at HSLL, including the sepsis code and earlier rule-based detection systems. Consequently, the observed reduction during the AI period should be interpreted as an overall improvement occurring during the implementation period, to which the AI system may have contributed, rather than as an effect exclusively attributable to AI. Although a difference in differences or synthetic control approach would have strengthened causal inference, no suitable contemporaneous control hospital with comparable cost data and without an AI-based sepsis system was available. Furthermore, while the COVID-19 period was excluded from the analysis, indirect post-pandemic changes in hospital practice may still have influenced the results.

The interpretation of the economic models also requires some caution. ICU admission and ICU readmission may partially mediate the relationship between AI-enabled early detection and hospital costs. For this reason, the primary generalized linear model, which adjusted for these variables, should be regarded as a conservative estimate of the direct effect, whereas the complementary model excluding them provides a broader estimate of the total effect. In addition, cost estimates were derived from standardized public payer reimbursement tariffs and therefore reflect average rather than marginal costs. As many hospital costs are fixed, reductions in length of stay may not translate into proportional short term cash savings, and freed capacity may subsequently be occupied by other patients. The reported ROI should therefore be interpreted as a long term estimate of the value associated with released capacity rather than as an immediate financial saving.

Generalisability may also be limited by the study setting. The analysis was conducted in a single tertiary hospital with a mature sepsis management infrastructure, including established escalation pathways, integrated electronic health records, and prior clinician familiarity with sepsis alerts. The magnitude of the observed benefits may therefore differ in hospitals with lower organizational readiness, in non-tertiary settings, or in healthcare systems operating under different reimbursement models.

Finally, the five year economic projections relied on assumptions regarding adoption rates, system performance, and cost behaviour. Although both deterministic and probabilistic sensitivity analyses, consistently demonstrated a positive net present value, the magnitude of the projected returns remains dependent on these assumptions. Moreover, the present study focused on economic and resource utilisation outcomes and did not evaluate clinical endpoints such as mortality, time to antibiotic administration, or clinical deterioration, which are being investigated separately. Similarly, clinician adoption, workflow adaptation, and alert fatigue were not directly assessed and warrant further study. Importantly, the lower ICU admission rate observed during the AI period could in principle reflect reduced quality of care, with patients who required intensive care being denied or delayed access. However, this interpretation is not supported by the available evidence. Lower-quality sepsis care would be expected to increase mortality, whereas the mortality of this unit, characterised in detail in a parallel 16-year analysis of the same hospital-wide protocol [20], shows no such deterioration. The concurrent reductions in 30-day and ICU readmissions observed here are also directionally inconsistent with under-triage. The most plausible interpretation is therefore not rationing of intensive care, but attenuation of sepsis severity through earlier recognition and treatment, whereby a proportion of patients who would have escalated to the ICU are instead managed on the ward.

## Conclusions

This real-world economic evaluation found that implementation of an AI-based sepsis prediction system at a tertiary hospital with mature sepsis infrastructure was associated with reductions in resource utilisation and hospital costs among 8,039 septic patients that were not fully explained by measured case-mix differences. The unadjusted, fully adjusted direct-effect, and total-effect GLM specifications all showed cost reductions in the AI implementation period. The direct-effect model estimated a 26.1% relative reduction in admission costs, while the total-effect model estimated a 31.1% reduction after adjustment for measured non-mediator confounders. The ITS analysis showed a borderline immediate level decrease at the time of AI deployment but no statistically significant slope change, against a backdrop of a pre-existing downward secular trend in costs. A more pronounced post-COVID downward slope is consistent with full AI integration but cannot be cleanly disentangled from post-pandemic normalisation. A 5-year economic simulation projected a cumulative discounted net benefit of approximately 3.55M€ and a positive NPV in the large majority of probabilistic simulations. Overall, the findings are consistent with AI implementation generating positive modelled economic value in this setting, while causal inference, generalisability to less-resourced hospitals, and the translation of average-cost savings into real-world cash flow remain limited by the quasi-experimental design.

## Supporting information

S1 File S1 Appendix. Interrupted time-series. S2 Appendix. Parameters base-case scenarios and sensitivity analysis. S3 Appendix. Total-effect gamma GLM (log link): Sensitivity model omitting ICU admission and ICU readmission. S4 Appendix. Pessimistic scenario: 5-year economic projection under lower system accuracy and slower adoption.
(DOCX)

## Abbreviations

AI: artificial intelligence
AUC: area under the curve
CHEERS-AI: Consolidated Health Economic Evaluation Reporting Standards for Artificial Intelligence
CI: confidence interval
DW: Durbin–Watson statistic
EHR: electronic health record
GLM: generalized linear model
HAC: heteroscedasticity- and autocorrelation-consistent
HSLL: Hospital Son Llàtzer
IB Salut: Balearic Islands Health Service
ICU: intensive care unit
IMV: invasive mechanical ventilation
IQR: interquartile range
ITS: interrupted time series
LOS: length of stay
LTE: limitation of therapeutic effort
ML: machine learning
NPV: net present value
OLS: ordinary least squares
PSA: probabilistic sensitivity analysis
qSOFA: quick Sequential Organ Failure Assessment
ROI: return on investment
SIRS: Systemic Inflammatory Response Syndrome
SOFA: Sequential Organ Failure Assessment
WHO: World Health Organization
