# Supplementary material for: Prospective economic evaluation of a predictive artificial intelligence model for sepsis: Effects on hospital costs and return on investment
Source: PLOS Glob Public Health. 2026 Aug 31;6(8):e0006059. doi: 10.1371/journal.pgph.0006059 (PMC13528994; doi:10.1371/journal.pgph.0006059)
Supplement: Supplementary file 1 [file pgph.0006059.s001.docx]

Supporting information

**S1 Appendix Interrupted Time-Series.**

<<Eqn11>>

<<Eqn12>>

**Where:**

• <<Eqn13>> is the mean cost per patient at time *t*.

• <<Eqn14>> is the baseline level of the series at *t* = 0.

• <<Eqn15>> represents the baseline (pre-intervention) trend.

• *I*_1_*_t_* is an indicator for the onset of the AI implementation.

• <<Eqn16>> captures the immediate level change at the onset of AI implementation.

• *T*_1_*_t_* counts the time elapsed since AI implementation.

• <<Eqn17>> represents the change in slope following AI implementation.

• *I*_2_*_t_* is an indicator for the onset of the COVID-19 period.

• <<Eqn18>> captures the immediate level change at the start of the COVID-19 period.

• *T*_2_*_t_* counts the time since COVID-19 onset.

• <<Eqn19>> represents the change in slope associated with the COVID-19 period.

• *I*_3_*_t_* is an indicator for the start of the post–COVID-19 period.

• <<Eqn20>> captures the immediate level change at the beginning of the post–COVID-19 period.

• *T*_3_*_t_* counts the time since the post–COVID-19 period began.

• <<Eqn21>> represents the change in slope in the post–COVID-19 period.

• <<Eqn22>> is the error term, assumed to be mean-zero with constant variance, and estimated using heteroscedasticity- and autocorrelation-consistent (HAC) Newey–West standard errors.

**S2 Appendix Parameters Base-Case Scenarios and Sensitivity Analysis**

Table 5. Base-case and deterministic sensitivity analyses relied on the mean parameter values. In the probabilistic sensitivity analysis, each parameter was modelled using an appropriate probability distribution, with its mean (<<Eqn23>>) derived from the economic model and its standard deviation (<<Eqn24>>) defined as a fixed proportion of the mean.

| **Parameter** | **<<Eqn25>>** | **<<Eqn26>>** | **Distribution** | **Source** |
| --- | --- | --- | --- | --- |
| <<Eqn27>> ICU days | 0.35 | 20% | Gamma | This study |
| <<Eqn28>> ward days | 0.59 | 20% | Gamma | This study |
| <<Eqn29>> ICU admissions | 0.04 | 25% | Beta | This study |
| Septic patient per year | 700 | 20% | Fixed | Hospital data |
| System accuracy | 0.85 | 0.05 | Beta | Vendor |
| Implementation cost | 200,000 | 40,000 | Gamma | Vendor |
| Annual maintenance cost | 100,000 | 20,000 | Gamma | Vendor |
| ICU day cost (€) | 2,094 | 20% | Gamma | Hospital tariffs |
| Ward day cost (€) | 1,243 | 20% | Gamma | Hospital tariffs |
| ICU Admission cost (€) | 8773 | 20% | Gamma | Hospital data |
| Discount rate (*r*) | 0.03 | — | Uniform(0.01–0.05) | Guidelines |
| AI Adoption ramp (%) | 60–80–80–100–100 | — | Fixed schedule | This study |

**S3 Appendix Total-Effect Gamma GLM (log link): Sensitivity Model Omitting ICU Admission and ICU Readmission**

To estimate the total effect of AI implementation on admission cost we fitted the Gamma GLM with log link after removing ICU admission and ICU readmission from the covariate set. All other variables were retained as in the primary specification. Full results are presented in Table 6.

Table 6. **Total-effect Gamma GLM (log link): sensitivity model without ICU admission and ICU readmission.**

| **Variable** | **Coef.** | **Std. Error** | **p-value** | **Exp(Coef.)** | **% Change** |
| --- | --- | --- | --- | --- | --- |
| Month (2) | 0.1170 | 0.069 | 0.088 | 1.124 | +12.4% |
| Month (3) | 0.0545 | 0.068 | 0.425 | 1.056 | +5.6% |
| Month (4) | 0.0886 | 0.071 | 0.214 | 1.093 | +9.3% |
| Month (5) | 0.0294 | 0.070 | 0.674 | 1.030 | +3.0% |
| Month (6) | 0.0480 | 0.074 | 0.515 | 1.049 | +4.9% |
| Month (7) | 0.0463 | 0.074 | 0.530 | 1.047 | +4.7% |
| Month (8) | 0.1258 | 0.076 | 0.096 | 1.134 | +13.4% |
| Month (9) | 0.1034 | 0.073 | 0.155 | 1.109 | +10.9% |
| Month (10) | 0.0543 | 0.073 | 0.457 | 1.056 | +5.6% |
| Month (11) | 0.0652 | 0.071 | 0.356 | 1.067 | +6.7% |
| Month (12) | 0.1079 | 0.069 | 0.118 | 1.114 | +11.4% |
| **AI implementation** | **-0.3728** | 0.055 | **<0.001** | **0.689** | **-31.1%** |
| **LTE** | **0.3806** | 0.041 | **<0.001** | **1.463** | **+46.3%** |
| **Age (years)** | **-0.0051** | 0.001 | **<0.001** | **0.995** | **-0.5%** |
| **Sex** | **-0.0966** | 0.031 | **0.002** | **0.908** | **-9.2%** |
| **Dysfunction** | **0.1009** | 0.011 | **<0.001** | **1.106** | **+10.6%** |
| **Year** | **0.0460** | 0.006 | **<0.001** | **1.047** | **+4.7%** |
| **Model summary** | | | | | |
| Number of observations | | | | 8,039 | |
| Residual degrees of freedom | | | | 8,021 | |
| Model degrees of freedom | | | | 17 | |
| Log-Likelihood | | | | -90,697 | |
| Deviance | | | | 7,856.6 | |
| Pearson chi-square | | | | <<Eqn30>> | |
| Pseudo *R*^2^ (Cragg–Uhler) | | | | 0.0369 | |

**S4 Appendix Pessimistic Scenario: 5-Year Economic Projection Under Lower System Accuracy and Slower Adoption**

Table 7. **Pessimistic scenario: annual economic impact under system accuracy = 0.65 and adoption ramp = 40–60–80–80–100%.**

| **Year** | **Benefits** | **Costs** | **Net** | **Disc.** | **Disc. Net** | **Cumulative** | **ROI** |
| --- | --- | --- | --- | --- | --- | --- | --- |
| 1 | 328,047 | 300,000 | 28,047 | 1.0000 | 28,047 | 28,047 | 9.3% |
| 2 | 492,071 | 100,000 | 392,071 | 0.9709 | 380,652 | 408,699 | 102.9% |
| 3 | 656,095 | 100,000 | 556,095 | 0.9426 | 524,173 | 932,872 | 189.9% |
| 4 | 656,095 | 100,000 | 556,095 | 0.9151 | 508,906 | 1,441,778 | 247.4% |
| 5 | 820,119 | 100,000 | 720,119 | 0.8885 | 639,816 | 2,081,594 | 309.9% |

Even under these simultaneously less favourable assumptions, the model projects a positive cumulative discounted net benefit from year 1 onwards, reaching 2,081,594€ (ROI 309.9%) by year 5. The reduction relative to the base case (3.55M€; ROI 528.2%) reflects the combined effect of lower per-patient savings scaling (accuracy 0.65 vs. 0.85) and a slower initial adoption ramp, which reduces cumulative benefits particularly in years 1 and 2. The positive sign of the net benefit under this scenario supports the robustness of the base-case conclusion to simultaneous pessimistic assumptions.
